# Methodology of Measuring Motoric Cognitive Risk Syndrome—Focusing on Slow Gait Speed: Protocol for a Systematic Review

**DOI:** 10.3389/fpsyt.2022.858950

**Published:** 2022-03-23

**Authors:** Liming Su, Xue Sun, Cheng Huang, Zhuqin Wei, Xinhua Shen, Lina Wang

**Affiliations:** ^1^School of Medicine, Huzhou University, Huzhou, China; ^2^Huzhou Third Municipal Hospital, The Affiliated Hospital of Huzhou University, Huzhou, China

**Keywords:** motoric cognitive risk syndrome, diagnostic criteria, subjective cognitive decline, slow gait speed, systematic review

## Abstract

**Introduction:**

Older adults with motoric cognitive risk (MCR) syndrome are at high risk of developing dementia. Although the definition of MCR is well recognized and consensus, previous studies did not reach an agreement on diagnostic criteria and measurement methods/tools for slow gait speed, which is one of four components of MCR diagnosis. The substantial heterogeneity in the methodology of slow gait speed diagnosis for MCR limits comparability and meta-analysis of studies.

**Objective:**

The study aims to conduct systematic and standardized integration for diagnostic criteria and methods of slow gait speed diagnosis for MCR based on previous evidence that may improve comparability between future studies.

**Methods:**

A systematic literature review will be undertaken by searching the following electronic databases (until February 1, 2022): PUBMED, EMBASE, The Cochrane Library, Web of Science. Additional studies will be identified by checking the reference lists of included studies or relevant reviews, manually searching the internet search engine Google Scholar, and searching the authors' personal files, if necessary. Two researchers will perform data extraction independently, and discrepancies will be resolved by discussion, which will include a third researcher if requires. The paper selection will perform in duplicate. Finally, a narrative account will synthesize the findings to answer the objectives of this review.

**Discussion:**

This is the first study on systematic and standardized integration for diagnostic criteria and measurement methods/tools for slow gait speed in diagnosing MCR. The findings of this study will be convenient for medical staff to examine the intended use and applicability of each instrument/tool for evaluating the gait speed, and provide insight into developing uniform guidelines for MCR.

**Systematic Review Registration:**

PROSPERO registration number: CRD42021232671.

## Introduction

### Rationale

Over 50 million people were estimated to be living with dementia globally in 2019, and this number is expected to increase to 152 million by 2050 (1, 2). However, effective treatments remain frustratingly elusive, and dementia has become an urgent challenge for the global community.

The current preclinical phase of dementia includes mild cognitive impairment (MCI) and motoric cognitive risk (MCR) syndrome (3, 4). MCR was a recently described predementia syndrome proposed by Verghese in 2013, characterized by cognitive complaints and slow gait speed without disability and dementia (5). Compared with MCI, MCR makes the assessment highly advantageous in detecting older adults at high risk of dementia (6, 7), avoiding the lengthy comprehensive neuropsychological evaluation and other time-consuming investigations (8, 9). It is more adaptable under cross-regional and cross-cultural backgrounds (5, 10, 11). Although the definition of MCR is well recognized and consensus (5, 10–15), previous studies did not reach an agreement on diagnostic criteria and measurement methods/tools for MCR, which would be a potential reason for a significant discrepancy in the prevalence of MCR in different studies, from 2–27.3% (10, 14, 16) all over the world (11, 17, 18). In general, the following four components have been proposed to be met for the diagnosis of MCR (5, 10): (a) subjective cognitive decline (SCD), (b) slow gait speed, (c) absence of dementia, and (d) independence in activities of daily living. Screening methods/tools for SCD, dementia and capability of daily living have been shown relative consistency in previous MCR studies, e.g., a question or a structured questionnaire about subjective memory loss was commonly used to screen SCD (10, 14, 19); the exclusion of dementia always refers to the diagnosis guidelines from ICD-10/11 or DSM-IV/V (5, 10, 15); the Activities of Daily Living (ADL) scale was applied to evaluate daily living capability. It is worth mentioning that slow gait speed is the critical diagnosis component of MCR; however, it always expresses controversy on diagnostic criteria and measurement methods/tools.

The evaluation of slow gait speed has significant controversy on the measurement tool, cut-off value, and length of walking required in different studies, including (1) A discrepancy of measurement tools: instrumented walkway with embedded pressure sensors (such as GAITRite ^®^, G-WALK ^®^) was commonly applied to evaluate gait speed in most recent studies (20–22). Besides, gait speed (m/s) was also calculated in the specific meter at the usual gait speed by a systematic stopwatch in several previous studies (23, 24). (2) A discrepancy of measurement length of walking: gait speed has been assessed through different length paths, ranging from 2.44 to 9.72 m (15, 23, 25, 26), allowing for the acceleration and deceleration phases. (3) A discrepancy of cut-off values for the diagnosis of slow gait speed: most of the studies considered slow gait as a binary variable using the different cut-off values, e.g., 0.8 m/s (27), 20th lower percentile (28), or ≥ 1 standard deviation (SD) below mean values of gait speed established within the same study cohort (18, 24, 29). Considering that gender and age might impact the diagnostic decisions of slow gait speed in the same cohort, some studies proposed that the diagnosis of slow gait speed should have age- and sex-appropriate cut-off value (27, 30). It is worth noting that a few studies have shown that gait speed might be difficult to assess during a primary care visit because of consultation rooms/space and consult time constraints (31, 32). In this context, a substitutive diagnosis component of MCR has been recommended, such as mobility performance using the five-times-sit-to-stand (FSTT) test (7, 33). Therefore, the most common measurement tool, length of walking, and cut-off value, for diagnosing slow gait speed in MCR individuals are unclear. In addition, whether better surrogate criteria for superseding slow gait speed remains to be elucidated.

The diagnostic criteria and measurement methods/tools for MCR are constantly evolving. There are no current uniform guidelines for MCR diagnosis, which impedes clinical practice and comparability of research on MCR across countries. Therefore, uniform diagnostic criteria and measurement methods/tools for MCR are needed. Given the scanty of studies on MCR diagnosis and the significant controversy on the diagnosis of slow gait speed, this study will conduct a systematic review to explore the ways used to diagnose MCR cases in all studies, using Verghese's criteria. It will focus on the variation of operationalization in the diagnosis component of slow gait speed for MCR.

### Objectives

This study aims to perform a systematic review and narrative synthesis of evidence on the methodology of measuring slow gait speed in diagnosing MCR.

## Methods

### Review Method

The protocol was registered with the International Prospective Register of Systematic Reviews (PROSPERO) database (registration number CRD42021232671) (34) on 22 February 2021, which is reported following the reporting guidance provided in the PRISMA-P statement (35, 36) (see PRISMA-P checklist in Supplementary Material 1).

### Patient and Public Involvement

No patients or the public will be involved in this study.

### Eligibility Criteria

Any original studies reporting the diagnostic criteria and measurement methods/tools for participants with MCR will be considered for inclusion in the final analysis.

Inclusion criteria will include the following: (1) The participant population included MCR; (2) Full text include MCR diagnostic criteria and measurement methods/tools.

Exclusion criteria will exclude the following: (1) Not relevant; (2) Not in English; (3) Inadequate description of slow gait speed methodology; (4) The full text does not include diagnostic criteria and measurement methods/tools for slow gait speed; (5) Duplicate analyses conducted on the same cohorts with the same assessment methods of slow gait speed; (6) Commentaries; (7) Methodology papers; (8) Conference abstracts; (9) Full text not available; (10) Animal or laboratory studies. Protocols, editorials, and letters will be excluded, but relevant references will be screened.

### Information Sources and Search Strategy

According to key terms from previous literature reviews and Medical Subject Headings (MeSH), the following electronic bibliographic databases will be searched (until February 1, 2022): PUBMED, EMBASE, The Cochrane Library, Web of Science. The literature search will be limited to the English language and human subjects. A detailed search strategy of PUBMED was built (see Table 1 in Supplementary Material 2), similar search strategies will be adapted to other electronic databases. The searches will be re-run before the final analysis, and additional studies will be retrieved.

To ensure literature saturation, we will check the reference lists of relevant studies, reviews, protocols, editorials, and letters identified by the search, and conduct a manual search of the internet search engine Google Scholar. In addition, if necessary, we will also search the authors' personal files to make sure that all relevant material has been captured. Finally, we will circulate a bibliography of the included studies to the systematic review team and experts identified by the team.

## Study Records

### Data Management

The abstracts and full-text articles retrieved from the search strategy will be imported into Endnote X9, and duplicates will be removed.

### Screening and Data Collection

**Figure 1:**
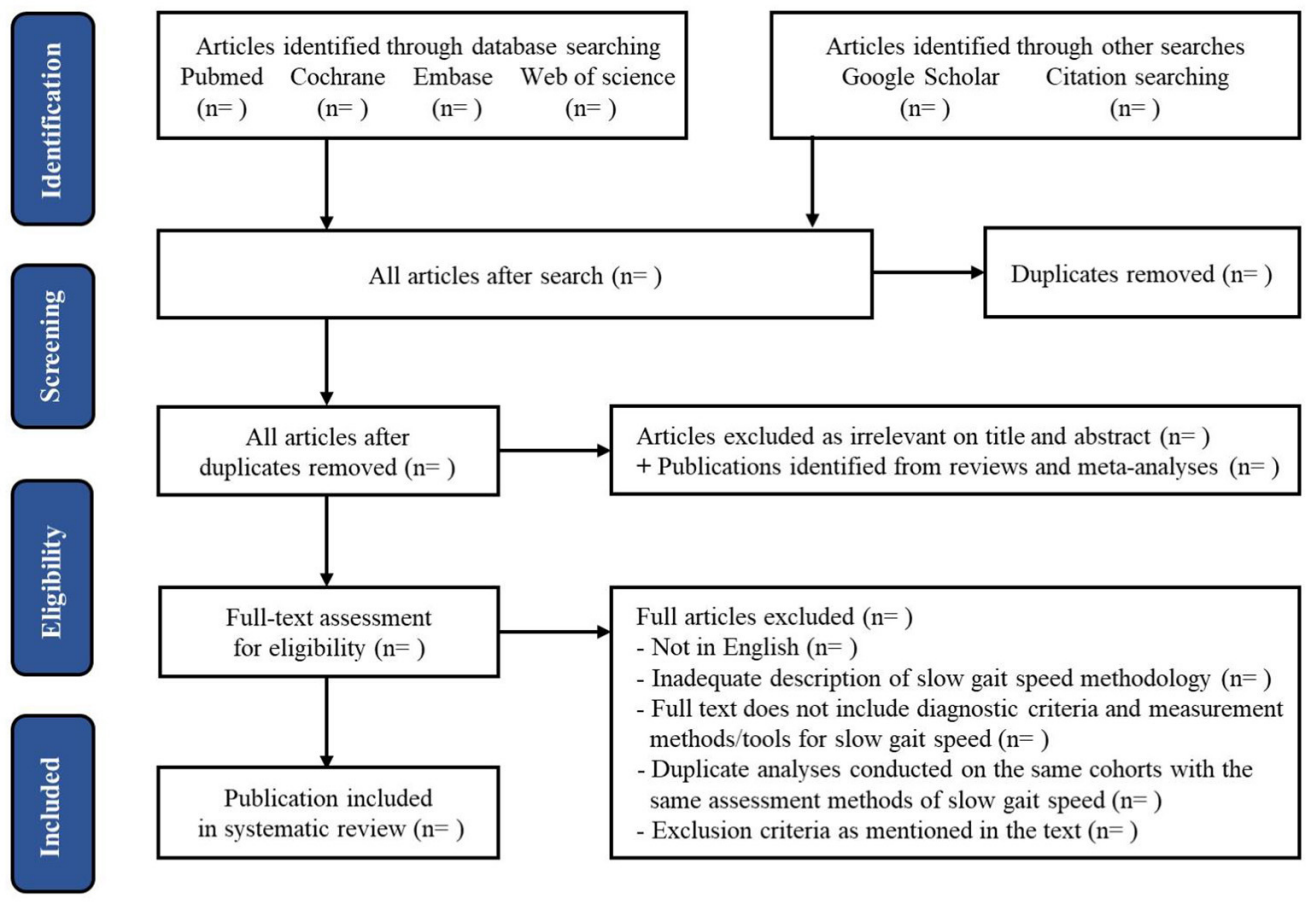
Systematic review flow-chart.

Two researchers will independently examine (1) titles and abstracts, and (2) full texts of relevant articles in two separate stages. Any study not meeting the inclusion criteria will be removed, and two researchers will resolve the disagreements by discussion or by a third researcher. Neither of the two researchers will be blind to the journal titles, the study authors, or institutions. According to the PRISMA, we will present the diagram of the selection process (37) (see Figure 1).

### Data Extraction

According to the previously designed and standardized sheet, two researchers will independently perform the data extraction from each eligible study. We will conduct calibration exercises before starting the review to ensure consistency across reviewers. The following data will be collected in the included studies: publication information, including the first author's name, publication year; study characteristics, such as country, research type, measurement setting, total study sample size; participant information, like age, number of the target population, prevalence, and inclusion and exclusion criteria; and screening information of MCR: except for SCD, dementia, and capability of daily living, the screening method of slow gait speed will be critical information for collection. This study will extract the cut-off values of slow gait in different age groups based on the gender stratification of the participants, and the cut-off value with the highest frequency of use will be considered “the most common cut-off value” for slow gait to match the particular gender and age. In cases where a publication characterized subjects using different MCR criteria, these criteria will be extracted separately and treated as separate entries for analysis (detail see Tables 2, 3 in Supplementary Material 2).

### Outcomes

This study will systematically review the differences in usage number of measurement methods/tools for screening slow gait speed or other potential surrogate criteria in diagnosing MCR, and further clarify the operationalization of slow gait speed criteria and the sex- and age-appropriate cut-off values to define gait slow in different countries and cohorts.

### Assessment of Risk of Bias

We are interested in the diagnostic criteria and measurement methods/tools of slow gait speed in diagnosing MCR in different studies, not the results of disease risk or treatment efficacy. Meanwhile, in terms of the scanty of studies on MCR diagnosis, we will conduct a purely narrative systematic literature review without an actual meta-analysis.

### Data Synthesis

A narrative synthesis will be conducted using tools and techniques informed by the Guidance on the Conduct of Narrative Synthesis in Systematic Reviews (38). We will comprehensively describe these research results and summarize the measurement methods/tools for slow gait speed or other potential surrogate criteria. A quantitative analysis will also be conducted to assess the difference in usage number of each diagnostic criteria/method and cut-off value, where appropriate, including the differences in the usage number of measurement tools for slow gait between measurement sites (clinical and community setting), and the differences in the usage number of cut-off values for slow gait based on the different characteristics of participants, like countries, genders, age groups. A chi-squared test or Fisher's exact test will be applied to verify the statistical significance of differences (P-value of < 0.05).

## Discussion

This study protocol follows the recommendations by the Preferred Reporting Items for Systematic Review and Meta-Analysis Protocols. Previous studies have shown that older adults with MCR are at high risk of developing dementia, including Alzheimer's disease with a two-fold increased risk and vascular dementia with a 12-fold increased risk (10, 27). One multicenter prevalence study reported that 20.3% of individuals with MCR could be reverted to non-MCR (11). Therefore, a rapid diagnosis of MCR is essential for dementia ultra-early prevention and intervention. Currently, there are inconsistent conclusions on diagnostic/measurement methods for MCR, especially for its important diagnosis component, i.e., gait slow speed, and no systematic review focusing on this topic. To the best of our knowledge, this is the first systematic review protocol on the comprehensive analysis of the methodology of measuring slow gait speed in diagnosing MCR. The findings of this review will provide the conceptual framework for the future development of MCR standardized diagnostic criteria and facilitate its consistent use in clinical practice and research.

Certain limitations in the design of this study should be noted. Language restriction to English might exclude additional studies published in other first languages, potentially introducing language and cultural biases. Furthermore, the scanty studies on MCR diagnosis will make meta-analysis difficult and sometimes impossible.

This protocol describes the integration framework of the diagnostic criteria and measurement methods/tools of slow gait speed in diagnosing MCR. The findings of this study will elaborate on the most common measurement tool, length of walking, and cut-off value, for diagnosing slow gait speed in MCR individuals. This study will provide critical clinical implications for the MCR screening practice. According to individual-level characteristics and screening space, medical staff will choose the appropriate measurement methods/tools of slow gait speed or better surrogate criteria for superseding slow gait speed. These matched-conditions measurement methods/tools of slow gait speed may provide insight into developing uniform guidelines for MCR.

### Amendments

Protocol amendments will be listed and made available on the PROSPERO registration. Any modifications to this protocol will be documented and published alongside the systematic review results.

## Author's Note

The study is currently preparing to search the literature. We will limit the date of the last literature retrieval to February 1, 2022, and we will re-run the literature search before the final analysis. This study will be completed in June 2022, and the timeline of the work is in detail as below:

- Complete the data extract at the end of Mar 2022;
- Complete the data analysis at the end of Apr 2022;
- Finish the manuscript and submit it at the end of June 2022.

## Ethics Statement

Ethical approval is not required for this systematic review as no primary data are collected. However, the findings of this study will be published in an open-access journal to ensure access for all stakeholders and disseminated during various scientific conferences.

## Author Contributions

LW and LS conceptualized the review. LS wrote the draft manuscript of this study. XS drafted the search strategies, CH and ZW will conduct article screening and data extraction. XS critically reviewed the protocol. LW is the guarantor. All authors contributed to the revision of the protocol. All authors have read and approved the final manuscript.

## Funding

This work was supported by the National Natural Science Foundation of China (Nos. 72174061 and 71704053), the China Scholarship Council Foundation (No. 201908330251), Zhejiang Provincial Natural Science Foundation of China (No. LQ17G030002), the General Scientific Research Project of Zhejiang Education Department (Y201942543), and the Zhejiang Provincial College Students Scientific and Technological Innovation Activities–Zhejiang Xinmiao Talents Program (No. 2021R431031).

## Conflict of Interest

The authors declare that the research was conducted in the absence of any commercial or financial relationships that could be construed as a potential conflict of interest.

## Publisher's Note

All claims expressed in this article are solely those of the authors and do not necessarily represent those of their affiliated organizations, or those of the publisher, the editors and the reviewers. Any product that may be evaluated in this article, or claim that may be made by its manufacturer, is not guaranteed or endorsed by the publisher.

## Abbreviations

MCR: Motoric Cognitive Risk Syndrome.
