# Supplementary material for: Methodology of Measuring Motoric Cognitive Risk Syndrome—Focusing on Slow Gait Speed: Protocol for a Systematic Review
Source: Front Psychiatry. 2022 Mar 23;13:858950. doi: 10.3389/fpsyt.2022.858950 (PMC8995464; doi:10.3389/fpsyt.2022.858950)
Supplement: Supplementary file 1 [file Data_Sheet_1.pdf]

Supplementary Material : Table 1-Table 3

Table 1 Search strategy of PUBMED

Number	Search term
1	motoric cognitive risk syndrome
2	motoric cognitive risk
3	cognitive motoric risk
4	motoric-cognitive
5	MCR
6	OR 1-5
7	"Walking speed"[MeSH]
8	walking speed
9	gait speed
10	gait pace
11	walking pace
12	gaits*
13	walk*
14	ambulation*
15	mobility*
16	OR 7-15
17	6 AND 16

Abbreviations:
- **MeSH,** Medical Subject Headings;
- **MCR,** motoric cognitive risk syndrome.

Table 2 Diagnostic criteria and methods of slow gait speed for MCR

Publication information		Study characteristics		Participant information			Slow gait speed			Surrogate criteria for slow gait speed	
First author	Year of publication	Country	Study type; Setting	N	Age, y (Mean ± SD)	Number of MCR; Prevalence of MCR, %	Screening method	Step-length	Cut-off value (m/s)	Screening method	Cut-off value

Table 3 The usage number distribution of the Measurement methods/tools of slow gait speed and other diagnosis components for MCR

Screening components	Measurement methods/tools	Number of studies	Study (First author, year)
**Slow gait speed**			
**Other components**			
SCD			
No dementia			
The capability of daily living			
